# Machine Learning Analysis Reveals Biomarkers for the Detection of Neurological Diseases

**DOI:** 10.3389/fnmol.2022.889728

**Published:** 2022-05-31

**Authors:** Simon Lam, Muhammad Arif, Xiya Song, Mathias Uhlén, Adil Mardinoglu

**Affiliations:** ^1^Centre for Host-Microbiome Interactions, Faculty of Dentistry, Oral & Craniofacial Sciences, King's College London, London, United Kingdom; ^2^Science for Life Laboratory, KTH—Royal Institute of Technology, Stockholm, Sweden

**Keywords:** systems biology, machine learning, neurodegeneration, GWAS—genome-wide association study, UK Biobank

## Abstract

It is critical to identify biomarkers for neurological diseases (NLDs) to accelerate drug discovery for effective treatment of patients of diseases that currently lack such treatments. In this work, we retrieved genotyping and clinical data from 1,223 UK Biobank participants to identify genetic and clinical biomarkers for NLDs, including Alzheimer's disease (AD), Parkinson's disease (PD), motor neuron disease (MND), and myasthenia gravis (MG). Using a machine learning modeling approach with Monte Carlo randomization, we identified a panel of informative diagnostic biomarkers for predicting AD, PD, MND, and MG, including classical liver disease markers such as alanine aminotransferase, alkaline phosphatase, and bilirubin. A multinomial model trained on accessible clinical markers could correctly predict an NLD diagnosis with an accuracy of 88.3%. We also explored genetic biomarkers. In a genome-wide association study of AD, PD, MND, and MG patients, we identified single nucleotide polymorphisms (SNPs) implicated in several craniofacial disorders such as apnoea and branchiootic syndrome. We found evidence for shared genetic risk loci among NLDs, including SNPs in cancer-related genes and SNPs known to be associated with non-brain cancers such as Wilms tumor, leukemia, and colon cancer. This indicates overlapping genetic characterizations among NLDs which challenges current clinical definitions of the neurological disorders. Taken together, this work demonstrates the value of data-driven approaches to identify novel biomarkers in the absence of any known or promising biomarkers.

## Introduction

Neurological diseases (NLDs) pose a significant public health problem due to aging populations and a widening global burden (Ray Dorsey et al., 2018; Nichols et al., 2019; Feigin et al., 2021). Currently, Alzheimer's disease (AD) is characterized by the accumulation of amyloid-β plaques and tau protein neurofibrillary tangles. These toxic species result in the destruction of cholinergic neurons and cause cognitive decline, leading to dementia. Parkinson's disease (PD) is characterized by α-synuclein inclusions in dopaminergic neurons in the substantia nigra, resulting in dopamine depletion and movement disorders. Motor neuron disease (MND) and myasthenia gravis (MG) are movement disorders that affect motor neurons and muscle, respectively, with MG also being an autoimmune disease. Individuals with PD or MND may also develop dementia.

Despite decades of research and clinical trials, there are currently no treatments to reverse the damage done by NLDs. Current research has focused on toxic species such as amyloid-β and tau in AD and α-synuclein in PD. Still, treatments derived from the research have been largely unsuccessful at clinical trials (Lam et al., 2020). Additional pharmacological and non-pharmacological targets need to be identified to enable early diagnosis and accelerate development of drugs and interventions to treat patients with NLDs effectively, and there is already great interest in this effort, revealing targets such as retinoid and androgen metabolism, YKL-40, AMIGO1, and GPRASP2; and suggesting that diet and lifestyle may also form part of an intervention (Toschi et al., 2019; Baldacci et al., 2020; Zool et al., 2020; Bayraktar et al., 2021; Lam et al., 2021).

**Figure 1:**
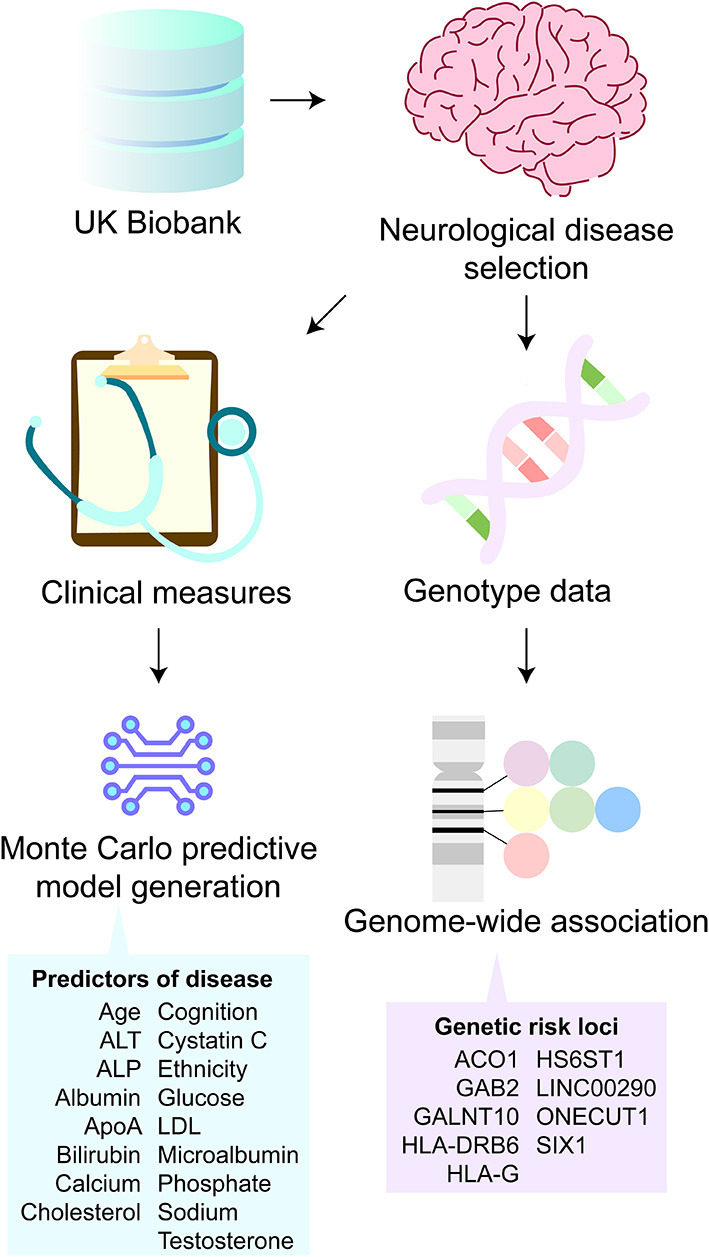
Study overview. From UK Biobank participants, Alzheimer's disease, Parkinson's disease, motor neuron disease (MND), and myasthenia gravis clinical and genotype data were obtained. The clinical data were used to train a predictive model of neurological diseases. The model was deconstructed to reveal predictors of disease relating to demographics, cognitive scores, and liver health biomarkers. The genotype data were used to perform genome-wide association studies. This analysis revealed nine genetic risk loci that were shared among all four neurological diseases.

In the present study, we retrieved clinical and genotyping data from 1,223 participants in the UK Biobank with NLDs (Sudlow et al., 2015), identified diagnostic markers for the predictions of NLDs, and identified shared genetic predispositions to NLDs (Figure 1). To this end, we used the clinical data to construct a predictive multinomial general linear model for AD, PD, MND, and MG and perform genome-wide association studies (GWAS). These diseases were chosen in order to represent a range of brain-related pathologies and a range of better-studied and less well-studied diseases.

The multinomial model was constructed using Monte Carlo randomization to select the optimal clinical variables to be included in the model and was able to predict NLD diagnosis with 88.3% accuracy. In addition to the promising true positive rate, inspection of the model weights correctly identified directional trends in blood and urine biochemistry and cognitive function that confirm previous reports in the literature. These included biomarkers which are normally associated with liver disease, such as alanine aminotransferase (ALT), alkaline phosphatase (ALP), and bilirubin, confirming the possibility of a role for the liver-brain axis in NLDs such as AD, as previously reported (Bassendine et al., 2020; Jakhmola-Mani et al., 2021).

We also used genotype data to identify genetic predispositions to NLDs. In GWAS, we found hundreds of risk alleles that were shared across at least two NLDs. There was enrichment for single nucleotide polymorphisms (SNPs) associated with non-brain cancers, such as Wilms tumor, leukemia, and colon cancer; and craniofacial syndromes, such as apnoea and branchiootic syndrome. We identified a panel of nine SNPs which were present in all four NLDs studied. These included two homeobox genes and three genes encoding biosynthetic enzymes. The identification of these shared SNPs suggests that the same SNP may be indicative of susceptibility to more than NLD and could potentially suggest that there exists a basal genetic perturbation that is linked with developing NLDs generally.

Taken together, our work demonstrates the usefulness of data-driven approaches to quickly identify easily measurable diagnostic biomarkers and shared genetic risk loci. Our results suggest that cognitive measures and biochemical markers usually associated with liver diseases are informative for the prediction of NLDs, and that SNPs linked with non-brain cancers and craniofacial disorders may be indicative of multiple NLDs. We therefore recommend this data-driven approach to guide future investigation for the study of diseases of unknown etiology or where promising biomarkers are yet unknown.

## Results

### Blood and Urine Biochemistry and Cognitive Trends Inform NLD Status

We retrieved clinical data from 1,223 patients with AD, PD, MND, or MG, of which 1,072 also had Affymetrix Axiom genotyping data on 820,967 SNPs, from the UK Biobank (Supplementary Table 1). The mean age of participants with AD, PD, MND, and MG was 62.8, 62.1, 59.4, and 60.2, respectively, compared with 53.7 for control participants. The male proportions were 56.6, 63.3, 69.2, and 46.6%, respectively, compared to 45.3% for controls. The cohort was made up of 89.5, 91.3, 90.8, and 91.4% British participants, respectively, compared with 87.1% British for controls.

**Figure 2:**
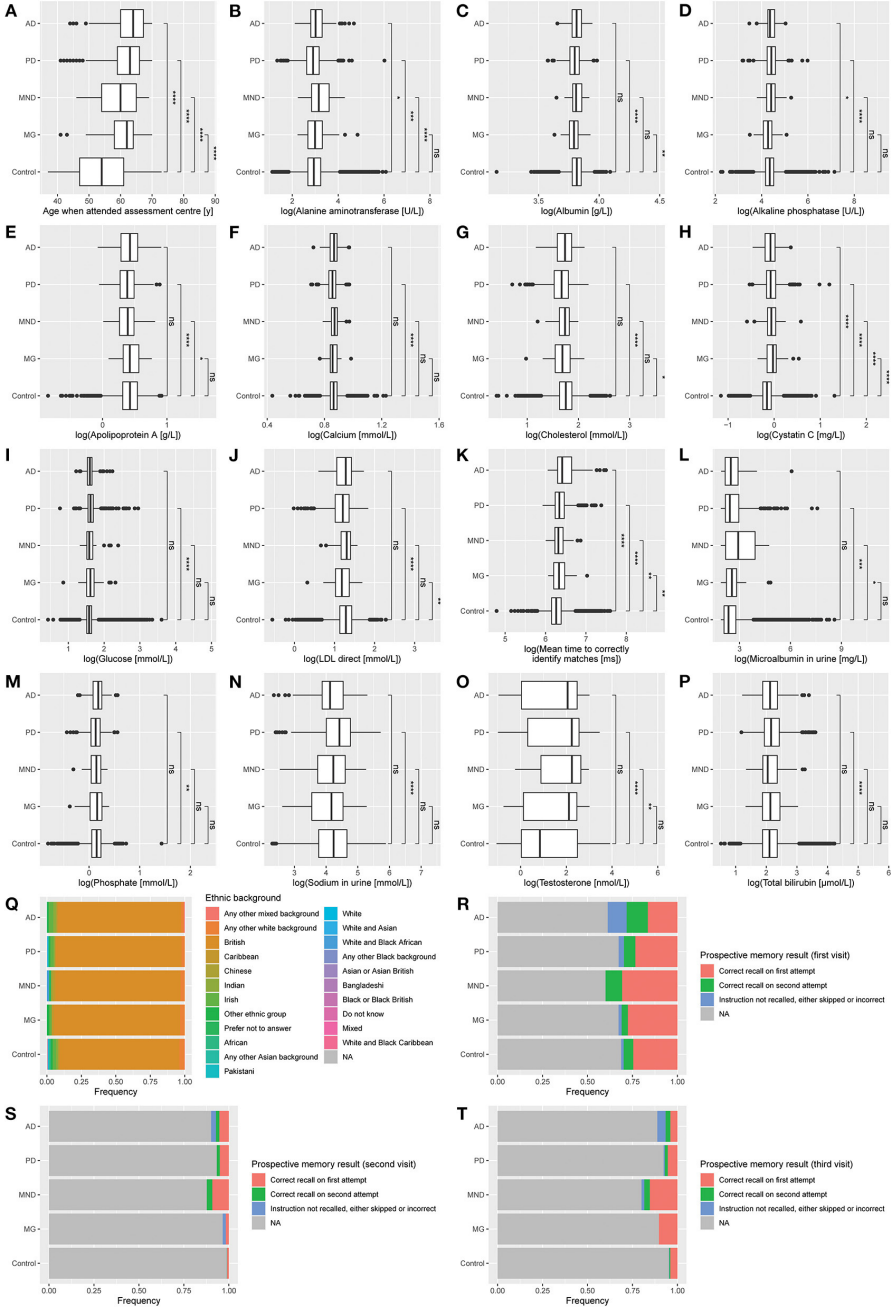
Distribution of clinical measures by diagnosis. **(A)** Age when attended assessment center. **(B)** Alanine aminotransferase. **(C)** Albumin. **(D)** Alkaline phosphatase. **(E)** Apolipoprotein A. **(F)** Calcium. **(G)** Cholesterol. **(H)** Cystatin C. **(I)** Glucose. **(J)** LDL direct. **(K)** Mean time to correctly identify matches. **(L)** Microalbumin in urine. **(M)** Phosphate. **(N)** Sodium in urine. **(O)** Testosterone. **(P)** Total bilirubin. **(Q)** Ethnic background. **(R)** Prospective memory result (first visit). **(S)** Prospective memory result (second visit). **(T)** Prospective memory result (third visit). Data represent all participants, including those whose samples which were not used in the training or testing of the multinomial model. AD, Alzheimer's disease (*n* = 152); PD, Parkinson's disease (*n* = 948); MND, motor neuron disease (*n* = 65); MG, myasthenia gravis (*n* = 58); Control (*n* = 116,559). Mean comparisons were performed using *t*-tests after log transformation (except for age). *, *p* ≤ 0.05; **, *p* ≤ 0.01; ***, *p* ≤ 0.001; ****, *p* ≤ 0.0001; ns, *p* > 0.05.

Patients with NLDs exhibited changes to blood and urine biochemical markers (Figure 2, Supplementary Table 2). Participants with AD had significantly higher ALT, ALP, and cystatin C levels and had the lowest performance in the cognitive tests. PD participants had lower ALT, albumin, apolipoprotein A (ApoA), calcium, cholesterol, low-density lipoprotein (LDL), and phosphate, but elevated ALP, bilirubin, glucose, microalbumin, sodium, and testosterone, compared to control. Participants with MND had significantly higher ALT, ALP, cystatin C, microalbumin, and testosterone compared to control, but lower ApoA. MG patients had decreased albumin, cholesterol, and LDL, but increased cystatin C compared to control. We noticed that a number of these clinical marker changes were involved in markers usually associated with liver disease. Indeed, this result is consistent with reports associating liver-based correlates with cognitive function (Kellett et al., 2011; Chen et al., 2021) and movement disorders (Lee and Yang, 2018; Jeong et al., 2021; Zhang et al., 2021).

All NLD patients took longer to correctly identify matches in cognitive testing than control. In the prospective memory test, AD patients were the most likely not to recall the instruction after two attempts and showed the least improvement between subsequent visits. This result was to be expected since AD is the disease most strongly associated with dementia and cognitive decline out of the four NLDs under investigation.

### A Multinomial Model Classified Neurological Diseases and Identified Relevant Biomarkers

From the empirical results suggesting clinical marker signatures in NLDs, we next sought to generate a predictive model of NLD diagnosis based on those markers. We therefore generated a multinomial generalized linear model to predict AD, PD, MND, MG, and control in a single test. We selected clinical measures with good coverage among participants to create this model and used Monte Carlo randomization to optimize the clinical measures to be included in the model (see Methods). The confounders in our final model can be summarized as follows: demographics (age, ethnicity), blood test measures (ALT, albumin, apolipoprotein A, calcium, cholesterol, cystatin C, LDL, phosphate, testosterone), urine test measures (microalbumin, sodium), and cognitive test measures (prospective memory test, reaction test). The multinomial model had a true positive rate of 88.3% on unseen data (Supplementary Table 3A).

We performed leave-one-out cross validation to demonstrate the essentiality of each of the variables included in the model in terms of its contribution to predictive power (Supplementary Table 4A). We constructed and tested new multinomial models after singly omitting each clinical marker, separately. We found that the model was robust to single variable omission, with most models performing at around 87–88% accuracy. The biggest drops arose from omission of prospective memory result (first visit) (76.0% accuracy), age (85.8% accuracy), and testosterone (87.0%). Omission of LDL, cystatin C, mean time to correctly identify matches, sodium, and apolipoprotein A all produced models with 87.7% accuracy. Age and cognition appeared to be the most important factors contributing to model accuracy.

We inspected the coefficients assigned to each of the clinical variables included in the model (Supplementary Table 5A). AD was more likely to be predicted for patients with low ALT, apolipoprotein A, cystatin C, LDL, and urine microalbumin. High calcium, cholesterol, and urine sodium were also characteristics of AD identified by the model. AD was the disease most tightly linked to advanced age, and patients of British, Chinese, or Irish background, were more likely to be predicted to have AD by the model, as did those who took the longest to identify matches. PD was assigned to those patients with advanced age, low ALT, low apolipoprotein A, and high testosterone. People of Chinese background or any other Asian background were less likely to be predicted to have PD. The model assigned MND to those patients with high ALT calcium, cholesterol, and testosterone, but low apolipoprotein A and phosphate. Patients who answered the ethnicity question with “any other white background” were more likely to be assigned MND by the model. The model linked MG with high ALT and cystatin C, but low albumin and LDL. Patients of British background were more likely to be assigned MG, whereas those of Irish and those who answered “any other white background” were less likely.

Across all diseases, the model applied a negative coefficient for the prospective memory test at the first visit, regardless of the result. Patients who took more attempts to recall correctly, or did not recall at all, were more likely to be assigned AD by the model than any other disease. Interestingly, the coefficients for PD were very similar regardless of the result. Together with the small coefficient given to the match identification test, the model suggests that cognitive measures were not very important for the classification of PD. In contrast, patients not recalling the instruction were given very negative coefficients for MND and MG, indicating that these patients are more likely to have a diagnosis of AD or PD instead.

Interestingly, at the second visit, the coefficients were always lower if the patient took two attempts rather than one, which appeared counter-intuitive for AD. However, AD also had the least negative coefficient, indicating that patients who took two attempts to recall the instruction at the second visit were more likely to be assigned AD.

The model also identified numerous interactions with blood and urine biochemical levels, indicating elevated ALT in MND and MG, but decreased in AD and PD; sharply decreased apolipoprotein A in AD and MND, but more modest decreases in PD and MG; increases in calcium and cholesterol in AD, MND, and MG, but changes in these molecules not being significant in PD; decreases in LDL and phosphate in any disease, but most strikingly in MG and MND, respectively; and increases in sodium and testosterone in all diseases. That the model was able to suggest these directional changes, which could not be so clearly concluded by *t*-tests alone (Figure 2), indicates the potential for machine learning techniques to identify possible biomarkers for classification in the absence of an understanding by the model of the underlying biology.

To determine whether biomarkers alone could predict NLD diagnoses, we repeated the Monte Carlo multinomial model but excluded all demographics data (that is, sex, age, and ethnic background) from the analysis. We found that this biomarker-only model was able to predict NLDs with an accuracy of 85.3%, which is comparable to the full model (Supplementary Table 3B), and indicates that much of the predictive power of the model could be explained by biomarkers alone. This is also consistent with the leave-one-out cross validation results on the full model (Supplementary Table 4A). The nine variables included in the model were alkaline phosphatase, glucose, mean time to correctly identify matches, urine microalbumin, phosphate, prospective memory result (first and third visits), testosterone, and total bilirubin. Similar to the full model, the biomarker-only model predicted NLDs on the basis of biomarkers normally associated with liver health (alkaline phosphatase, glucose, and total bilirubin).

In addition, inspecting the model weights in the biomarker-only model revealed retained predicted effects of clinical measures on NLD prediction (Supplementary Table 5B). The biomarker-only model introduced four clinical measures that were not selected in the full model. Alkaline phosphatase was predictive for AD when elevated but for MG when depleted, and glucose was predictive for MG when elevated but AD when depleted. Prospective memory test (third visit) was predictive for NLDs in a similar manner to second visit results in the full model. Total bilirubin was also predictive for AD when depleted.

In leave-one-out cross validation of the biomarker-only model, model accuracy was not impacted by single removal of alkaline phosphatase, glucose, urine microalbumin, or phosphate as predictors (Supplementary Table 4B). All other predictors only modestly reduced the model accuracy from 85.3 to 85.1%, with the exception of prospective memory result (first visit), whose removal resulted in a model accuracy of only 66.9%. This is in line with the full model and suggests that simple cognitive tests are highly informative when diagnosing NLDs.

### Genome-Wide Association Uncovered Shared Heritable Factors Between Neurological Diseases

**Figure 3:**
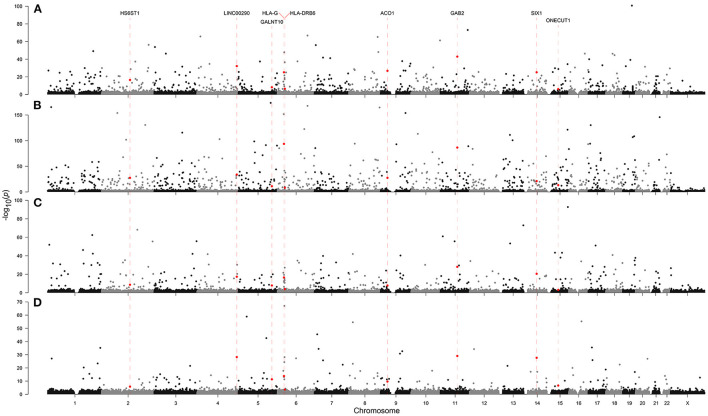
Genome-wide association with **(A)** Alzheimer's disease, **(B)** Parkinson's disease, **(C)** motor neuron disease, and **(D)** myasthenia gravis in the Affymetrix Axiom Biobank Array. Dashed red lines and red points indicate SNPs which appeared in the top 1,000 associations by asymptotic Cochran-Armitage trend test *p*-value in all four neurological diseases.

**Figure 4:**
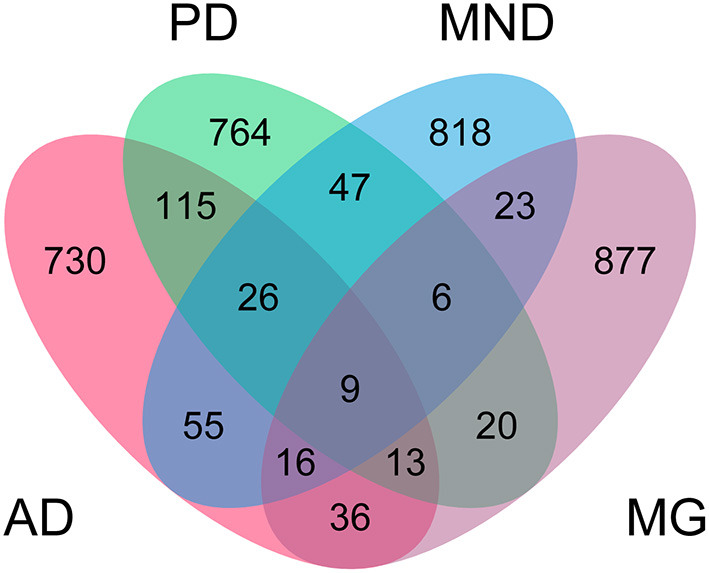
Distribution of shared SNPs across neurological diseases. The top 1,000 SNPs identified by genome-wide association study with Alzheimer's disease (AD), Parkinson's disease (PD), motor neuron disease (MND), and myasthenia gravis (MG) were inspected for overlap across multiple diseases. Numbers indicate number of SNPs shared between diseases.

In the multinomial model, we identified a heritable variable, ethnicity, to predict neurodegeneration. Therefore, we hypothesized that there might be more heritable factors that could alter a person's susceptibility to NLDs. To identify heritable factors, we analyzed genotyping data originating from an Affymetrix Axiom array, which covers 836,727 SNPs (Figures 3, 4, Supplementary Figure 1, Supplementary Table 6). We found significant SNPs which were also linked with non-brain cancers and craniofacial disorders. Many of these SNPs were found to be associated with more than one NLD. For example, AD, PD, and MND were all associated with SNPs in LAMA2, PTPN12, and SPATA7, which are implicated in muscular dystrophy, colon cancer, and retinitis pigmentosa, respectively. AD, PD, and MG all had SNPs in PTCH1 (associated with holoprosencephaly) and XKR9 (associated with otofaciocervical syndrome). AD, MND, and MG were all associated with SNPs in VPS41 (associated with Wilms tumor) and CHRM3 (associated with Eagle-Barrett syndrome). In total, we found 70 SNPs linked with at least three of the four NLDs under investigation.

Considering the functions of genes affected by the SNPs we identified, we found a vast number of significant SNPs within genes associated with cancer, its hallmarks, or neurotransmission (Supplementary Data 1). An intronic SNP in the scaffold protein interactor GAB2 was associated with all four diseases. A missense mutation in the TNFα response gene TNFAIP3 gene was highly associated with AD and PD, but not MND or MG. AD was also associated with SNPs in the cytochrome P450 gene CYP2B6, adenylate cyclase ADCY8, and signaling gene PIK3C3 (PI3K). PD was highly associated with SNPs in dysferlin (DYSF), TP53, and numerous cytoskeleton genes such as AFAP1L1 and MYH1. MND was associated with SNPs in retinoblastoma protein interactor RBBP5, tumor necrosis factor family member TNFRSF25, and several cytoskeleton function genes, including tropomyosin TPM1 and troponin TNNI3. MND was also associated with SNPs in spermatogenesis associated genes such as SPATA8 and SPAG16. MG was associated with SNPs in hallmarks of cancer–related genes such as the microtubule-associated tumor suppressor MTUS1, leukocyte-associated gene LAIR2, and Cal proto-oncogene CBLB. MG was also associated with SNPs in the brain- and neuron-specific genes, including cerebellum 4 precursor (CBLN4), potassium channel KCNH5, adenylate cyclase 8 (ADCY8), and autism susceptibility candidate gene AUTS2.

In addition to GAB2, we also identified a further eight SNPs which were associated with all four diseases (Figure 4, Supplementary Table 6). LINC00290, ACO1, HLA-G, SIX1, HS6ST1, GALNT10, ONECUT1, and HLA-DRB6 were all present in the top 1,000 SNP associations by asymptotic *p*-value in all four diseases. Of these, HLA-G, SIX1, HS6ST1, ONECUT1, and HLA-DRB6 have existing annotations in the Online Mendelian Inheritance in Man (OMIM) database related to brain and craniofacial syndromes. ACO1, HS6ST1, and GALNT10 encode biosynthetic enzymes, strengthening our result in the multinomial models suggesting a role for biochemistry-based biomarkers. SIX1 and ONECUT1 are homeobox genes. SIX1 is a master regulator in multiple tissues and ONECUT1 is a transcription factor of the liver; and are associated with the craniofacial disorders branchiootic syndrome and amelogenesis imperfecta, respectively. This further strengthens the proposed link between NLDs and other tissues, and suggests that interactions with other tissues may also influence craniofacial disorders, but further investigation is required to confirm this.

These results indicate that the same SNPs may be associated with susceptibility to more than one NLD. Further, the identification of NLD risk SNPs linked with cancer risk is significant as it is consistent with the reported degeneration/cancer antagonistic shift (Aramillo Irizar et al., 2018). The additional identification of NLD risk SNPs linked with craniofacial disorders suggests a potentially interesting axis of investigation for NLDs which is not well explored at present (Kamer et al., 2020). Taken together with the results from multinomial modeling, we propose a paradigm shift in NLD biomarker identification to focus on liver biochemistry, interactions with non-brain cancer, and association with craniofacial disorders.

## Discussion

This study shows the value of big biological data in driving hypothesis-free studies toward the early diagnosis and prediction of genetic risk loci in NLDs. Inspired by empirical trends in clinical marker data (Figure 2, Supplementary Table 2), we first constructed a multinomial model that could predict AD, PD, MND, or MG with an accuracy of 88.3% (Supplementary Table 3). The clinical variables that we used to generate the model were selected by a Monte Carlo randomization method, and the model weightings could be used to direct future NLD research.

Regarding the multinomial model, deconstruction of the model weightings revealed directional machine-learned associations between clinical markers and diseases (Supplementary Table 5). Some of these associations have already been reported in the literature: for instance, our model predicted low ALT in AD, which is consistent with the literature (Lu et al., 2021). Our model also correctly predicted low apolipoprotein A in AD, PD, and MND (Qiang et al., 2013; Mariosa et al., 2017; Zuin et al., 2021) and elevated calcium in AD and PD (Zakharov et al., 2007; Angelova et al., 2016; Ryan et al., 2020). Such model deconstruction after machine learning therefore appears to be a useful tool for identifying biomarkers and guiding further investigation in a manner that is agnostic to the assumptions of the underlying biology.

Leave-one-out cross validation (Supplementary Table 4A) on the model and assessment of non-demographic clinical markers only (Supplementary Tables 3B, 4B, 5B) revealed that cognitive scores were the single most important variable in the model in terms of predictive power. Single removal of any other variable from the model did not adversely impact the predictive power of the model, indicating that no single variable (apart from cognitive scores) could reasonably predict NLDs. This, along with the Monte Carlo randomized selection of biomarkers usually attributed to liver health, represents a paradigm shift away from single species in NLD research such as amyloid-β in AD and α-synuclein in PD.

Having also included ethnicity in the full multinomial model, we next carried out a GWAS to identify other heritable factors which might predispose an individual to NLDs (Figures 3, 4). While we could not account for the variability in the data due to ethnicity, we found commonalities across all four diseases—particularly the appearance of SNPs associated with non-brain cancers and craniofacial disorders. The identification of hundreds of SNPs shared across at least two NLDs, dozens of SNPs shared across at least three NLDs, and nine SNPs shared across all four NLDs strikingly suggests some common but yet unknown genetic mechanism associated with NLD in general. We found a marked enrichment of SNPs annotated in the OMIM database to be linked with non-brain cancers and craniofacial disorders, as well as three SNPs in biosynthetic genes (ACO1, HS6ST1, and GALNT10) and two SNPs in homeobox genes (SIX1 and ONECUT1) among the nine SNPs shared by all four NLDs. Our GWAS results therefore suggest that the same SNPs may confer susceptibility to more than one NLD, and may also represent a risk factor for non-brain cancer and craniofacial disorders. More research must be conducted to uncover the genetic mechanism that appears to be common to all of these conditions.

It is important to acknowledge the limitations of the study. Firstly, the multinomial model weightings contradicted the literature in some places. An example of this was the significant negative coefficient for LDL in MND, suggesting that MND patients might have lower serum LDL, whereas this is reported not to be the case (Chen et al., 2018; Zeng and Zhou, 2019). Secondly, although the model had an accuracy of 88.3%, it predicted some diseases better than others. Both of these limitations likely were due to the relatively low numbers of patients with AD, MND, and MG compared to PD and control. Because of this, we indicate that the model may be improved with the inclusion of more samples and more consistent data collection across all patients. This limitation, however, did not detract from the model's usefulness to be deconstructed to predict biomarkers, and does not change the fact that any predictions from the model must be treated as a starting point for future validation. Thirdly, the model did not include any biomarkers from cerebrospinal fluid (CSF). Cerebrospinal fluid measures are potentially informative due to circulating factors which are not detectable in blood. However, the UK Biobank does not collect such data, so we could not analyses these factors in the current study. Regarding the GWAS, again, our investigation would have benefited with more even patient numbers across NLDs. The studies conducted on AD and PD samples were more powerful than those on MND and MG. We overcame this limitation by inspecting the top 1,000 SNPs per NLD rather than imposing a *p*-value cutoff, which also controlled false positive discoveries in the larger AD and PD patient pools.

In conclusion, we propose data-driven machine learning and data exploration by GWAS as ideal first steps toward biomarker discovery for diseases of unknown etiology or currently lacking promising biomarkers. Such data-driven approaches may be extended to bench experimental work and are expected to guide dynamic detection and quantification of target druggability, *in vivo* demonstration of mechanisms of action, and prediction of drug resistance mechanisms. In this work, we demonstrated the value of a data-driven approach by identifying accessible blood, urine, and cognitive biomarkers in AD, PD, MND, and MG using a machine learning multinomial model with no knowledge of the underlying biology. This approach highlighted liver enzymes as potentially diagnostic biomarkers for NLDs and should now be targets for basic biology research in NLDs as outlined above. We also used GWAS to confirm shared genetic risk loci between NLDs, cancer, and craniofacial disorders. Although the aging-related antagonistic switch between degeneration and cancer has been reported, the association between NLD and craniofacial disorders is a yet-untapped arena that demands further investigation.

## Methods

### Acquisition of Data and Inclusion Criteria

Data were obtained from UK Biobank (Sudlow et al., 2015). Samples were accepted as AD, MG, MND, or PD samples if any of the respective diseases were recorded by UK Biobank, even if other conditions were also recorded. UK Biobank records conditions and diseases on a self-reporting basis. Participants are asked to self-report by answering the question in a UK Biobank questionnaire: “You selected that you have been told by a doctor that you have other (non-cancer) serious illnesses or disabilities, could you now tell me what they are?” Control samples were accepted as those without any recorded h diseases (Supplementary Table 1).

### Generation of the Multinomial Model and Leave-One-Out Cross Validation

To generate the multinomial model, standard and easily-measurable clinical data including demographics, sight and hearing problems, diabetes diagnosis, stroke diagnosis, medication and treatment, illness, operations, cognitive and mental measures, brain measurements, blood and urine tests, and adverse events and death were obtained from UK Biobank. Clinical variables were removed from the analysis if <75% of samples had a value recorded. Categorical measures with only one category were also removed. After quality control, 40 clinical variables remained in the analysis. A separate model was also generated excluding demographic data.

Monte Carlo randomization was used to randomly sample clinical variables as independent variables in a multinomial model. Samples with any missing values across the selected clinical variables were dropped. The multinomial model was constructed using the R nnet package (version 7.3-14, https://cran.r-project.org/web/packages/nnet/index.html, accessed 2021-06-01). The dependent variable was disease. The dataset was randomly split into training (70%) and test sets (30%) to assess the accuracy (true positive rate) of the model on test set data. The model with the highest accuracy after 1,000 random samplings was accepted for further consideration.

To find the model with the local maximum accuracy, clinical variables not in the model were added, and clinical variables already in the model were removed, individually. If, after each model change, the model's accuracy improved, then that change was kept; if otherwise, then that change was reverted.

To perform leave-one-out cross validation on the multinomial model, variables were singly removed from the model, separately, and multinomial models were constructed and tested, as above.

### Genotyping Analysis

Raw genome-wide genotyping data were obtained from the UK Biobank Axiom Array. Axiom Analysis Suite (version 5.1.1, ThermoFisher, accessed 2021-06-01) was used to perform quality control and analysis on the raw data to determine patient genotypes at each SNP, using the Best Practices Workflow. Allele frequencies were computed from genotype data for each disease class. To detect significant differences in allele frequencies between disease and control, Cochran-Armitage trend tests were performed assuming a codominant allele model. The test statistics, exact *p*-values, and asymptotic *p*-values were recorded. CATTexact (version 0.1.1, https://cran.r-project.org/web/packages/CATTexact/index.html, accessed 2021-06-01) R package was used for the computation of Cochran-Armitage *p*-values (Mehta et al., 1992). qqman (version 0.1.8, https://cran.r-project.org/web/packages/qqman/index.html, accessed 2021-06-01) R package was used to plot the Manhattan and quantile-quantile plots. The top 1,000 SNPs by asymptotic *p*-value for each NLD were accepted for further visualization in a Venn diagram. ggvenn (version 0.1.9, https://cran.r-project.org/web/packages/ggvenn/index.html, accessed 2022-03-31) R package was used to plot the Venn diagram. All bioinformatic and statistical analyses were performed in R (version 4.0.2) unless otherwise indicated.

## Data Availability Statement

The datasets presented in this study can be found in online repositories. The names of the repository/repositories and accession number(s) can be found below: https://github.com/SimonLammmm/ukbb-ndd-ml.

## Ethics Statement

Ethical review and approval was not required for the study on human participants in accordance with the local legislation and institutional requirements. Written informed consent for participation was not required for this study in accordance with the national legislation and the institutional requirements.

## Author Contributions

MU and AM supervised the study. SL designed the study, performed all the analyses, and analyzed all the results. SL wrote the manuscript with input from MA, XS, MU, and AM. All authors contributed to the article and approved the submitted version.

## Funding

This work was financially supported by Knut and Alice Wallenberg Foundation (Grant No. 2017.0303) to AM.

## Conflict of Interest

The authors declare that the research was conducted in the absence of any commercial or financial relationships that could be construed as a potential conflict of interest.

## Publisher's Note

All claims expressed in this article are solely those of the authors and do not necessarily represent those of their affiliated organizations, or those of the publisher, the editors and the reviewers. Any product that may be evaluated in this article, or claim that may be made by its manufacturer, is not guaranteed or endorsed by the publisher.
