# Supplementary figures and images for: Machine Learning Analysis Reveals Biomarkers for the Detection of Neurological Diseases
Source: Front Mol Neurosci. 2022 May 31;15:889728. doi: 10.3389/fnmol.2022.889728 (PMC9194858; doi:10.3389/fnmol.2022.889728)

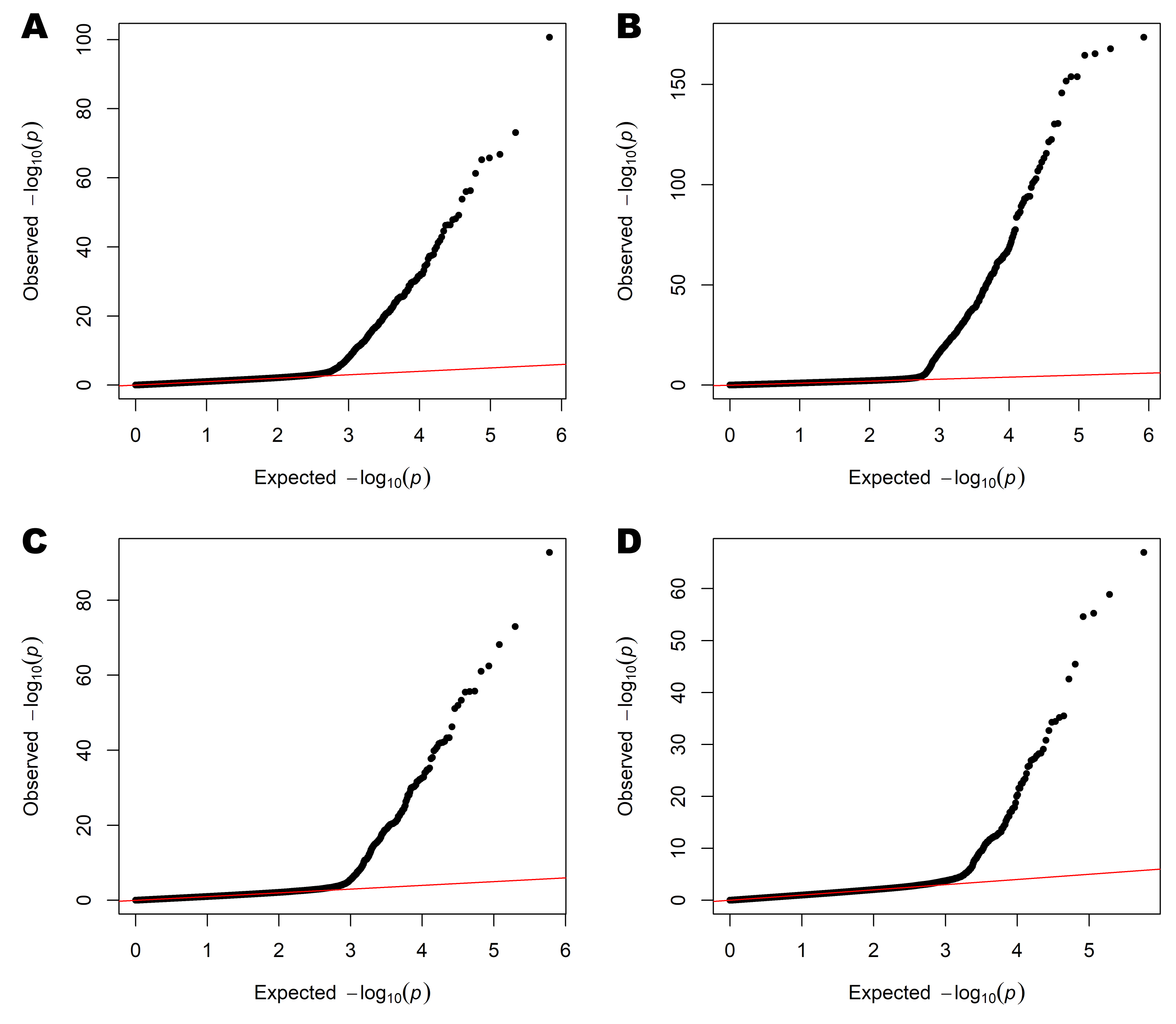

Supplement: Supplementary file 2 [file Image_1.TIF]
