# Supplementary material for: Machine Learning Analysis Reveals Biomarkers for the Detection of Neurological Diseases
Source: Front Mol Neurosci. 2022 May 31;15:889728. doi: 10.3389/fnmol.2022.889728 (PMC9194858; doi:10.3389/fnmol.2022.889728)
Supplement: Supplementary file 3 [file Table_1.DOCX]

Supplementary Table 1. Characteristics of UK Biobank NDD dataset

| Disease | Total | With genotyping data |
| --- | --- | --- |
| AD | 152 | 129 |
| PD | 948 | 832 |
| MND | 65 | 60 |
| MG | 58 | 51 |
| Control | 116559 | 446 |
| Total (excluding controls) | 1223 | 1072 |
