# Supplementary material for: Machine Learning Analysis Reveals Biomarkers for the Detection of Neurological Diseases
Source: Front Mol Neurosci. 2022 May 31;15:889728. doi: 10.3389/fnmol.2022.889728 (PMC9194858; doi:10.3389/fnmol.2022.889728)
Supplement: Supplementary file 4 [file Table_2.DOCX]

Supplementary Table 2. Summary statistics of UK Biobank NDD dataset

| Clinical marker | Disease | Mean | 95% CI | | p-value (t-test)* |
| --- | --- | --- | --- | --- | --- |
| **Age when attended assessment centre [y]** | AD | 62.77 | 61.86 | 63.68 | 2.17 × 10^-43^ |
|  | PD | 62.13 | 61.79 | 62.48 | 1.80 × 10^-255^ |
|  | MND | 59.40 | 57.78 | 61.02 | 1.92 × 10^-9^ |
|  | MG | 60.17 | 58.50 | 61.84 | 2.12 × 10^-10^ |
|  | Control | 53.74 | 53.69 | 53.78 |  |
| **Alanine aminotransferase [U/L]** | AD | 24.06 | 21.81 | 26.31 | 0.030 |
|  | PD | 20.81 | 19.75 | 21.87 | 1.20 × 10^-5^ |
|  | MND | 28.21 | 24.80 | 31.62 | 1.82 × 10^-4^ |
|  | MG | 24.59 | 19.68 | 29.50 | 0.402 |
|  | Control | 22.07 | 21.99 | 22.14 |  |
| **Albumin [g/L]** | AD | 45.30 | 44.87 | 45.74 | 0.571 |
|  | PD | 44.64 | 44.47 | 44.81 | 8.21 × 10^-17^ |
|  | MND | 45.40 | 44.81 | 45.99 | 0.951 |
|  | MG | 44.41 | 43.74 | 45.07 | 5.61 × 10^-3^ |
|  | Control | 45.43 | 45.42 | 45.45 |  |
| **Alkaline phosphatase [U/L]** | AD | 4.40 | 4.36 | 4.44 | 0.014 |
|  | PD | 4.42 | 4.40 | 4.44 | 1.07 × 10^-13^ |
|  | MND | 4.42 | 4.35 | 4.49 | 0.044 |
|  | MG | 4.28 | 4.20 | 4.37 | 0.146 |
|  | Control | 4.35 | 4.34 | 4.35 |  |
| **Apolipoprotein A [g/L]** | AD | 1.55 | 1.50 | 1.59 | 0.531 |
|  | PD | 1.48 | 1.47 | 1.50 | 4.53 × 10^-16^ |
|  | MND | 1.47 | 1.41 | 1.53 | 0.0144 |
|  | MG | 1.58 | 1.51 | 1.65 | 0.590 |
|  | Control | 1.56 | 1.56 | 1.56 |  |
| **Calcium [mmol/L]** | AD | 2.38 | 2.36 | 2.39 | 0.977 |
|  | PD | 2.35 | 2.35 | 2.36 | 1.21 × 10^-13^ |
|  | MND | 2.39 | 2.37 | 2.42 | 0.225 |
|  | MG | 2.36 | 2.34 | 2.39 | 0.255 |
|  | Control | 2.38 | 2.38 | 2.38 |  |
| **Cholesterol [mmol/L]** | AD | 5.70 | 5.52 | 5.88 | 0.0814 |
|  | PD | 5.37 | 5.30 | 5.44 | 2.92 × 10^-33^ |
|  | MND | 5.64 | 5.42 | 5.85 | 0.115 |
|  | MG | 5.48 | 5.17 | 5.80 | 0.0203 |
|  | Control | 5.85 | 5.84 | 5.86 |  |
| **Cystatin C [mg/L]** | AD | 0.94 | 0.91 | 0.96 | 1.03 × 10^-7^ |
|  | PD | 0.96 | 0.94 | 0.97 | 8.85 × 10^-52^ |
|  | MND | 0.96 | 0.91 | 1.00 | 3.22 × 10^-4^ |
|  | MG | 0.99 | 0.94 | 1.04 | 2.92 × 10^-6^ |
|  | Control | 0.87 | 0.87 | 0.87 |  |
| **Glucose [mmol/L]** | AD | 1.61 | 1.59 | 1.63 | 0.056 |
|  | PD | 1.64 | 1.63 | 1.65 | 3.76 × 10^-17^ |
|  | MND | 1.62 | 1.58 | 1.67 | 0.165 |
|  | MG | 1.64 | 1.58 | 1.70 | 0.112 |
|  | Control | 1.59 | 1.59 | 1.59 |  |
| **LDL direct [mmol/L]** | AD | 3.57 | 3.43 | 3.71 | 0.125 |
|  | PD | 3.36 | 3.30 | 3.41 | 1.50 × 10^-25^ |
|  | MND | 3.57 | 3.41 | 3.72 | 0.399 |
|  | MG | 3.34 | 3.12 | 3.57 | 6.54 × 10^-3^ |
|  | Control | 3.67 | 3.66 | 3.67 |  |
| **Mean time to correctly identify matches [ms]** | AD | 693.30 | 654.25 | 732.35 | 5.15 × 10^-14^ |
|  | PD | 591.66 | 583.36 | 599.96 | 2.99 × 10^-34^ |
|  | MND | 579.71 | 551.68 | 607.74 | 6.22 × 10^-3^ |
|  | MG | 588.43 | 555.41 | 621.45 | 3.54 × 10^-3^ |
|  | Control | 544.18 | 543.55 | 544.81 |  |
| **Microalbumin in urine [mg/L]** | AD | 24.03 | 15.01 | 33.05 | 0.212 |
|  | PD | 28.70 | 21.01 | 36.39 | 4.04 × 10^-3^ |
|  | MND | 31.02 | 23.76 | 38.28 | 0.0314 |
|  | MG | 23.48 | 15.09 | 31.87 | 0.261 |
|  | Control | 20.92 | 20.47 | 21.38 |  |
| **Phosphate [mmol/L]** | AD | 1.18 | 1.16 | 1.21 | 0.106 |
|  | PD | 1.14 | 1.13 | 1.15 | 7.17 × 10^-3^ |
|  | MND | 1.14 | 1.11 | 1.18 | 0.566 |
|  | MG | 1.15 | 1.10 | 1.20 | 0.619 |
|  | Control | 1.16 | 1.16 | 1.16 |  |
| **Sodium in urine [mmol/L]** | AD | 74.39 | 67.77 | 81.02 | 0.447 |
|  | PD | 89.43 | 86.57 | 92.29 | 6.70 × 10^-19^ |
|  | MND | 74.53 | 64.05 | 85.01 | 0.562 |
|  | MG | 75.38 | 63.24 | 87.51 | 0.466 |
|  | Control | 78.48 | 78.22 | 78.74 |  |
| **Testosterone [nmol/L]** | AD | 7.19 | 6.29 | 8.10 | 0.114 |
|  | PD | 8.47 | 8.08 | 8.85 | 1.86 × 10^-20^ |
|  | MND | 8.83 | 7.38 | 10.28 | 1.53 × 10^-4^ |
|  | MG | 7.63 | 6.05 | 9.21 | 0.174 |
|  | Control | 6.66 | 6.62 | 6.70 |  |
| **Total bilirubin [µmol/L]** | AD | 2.14 | 2.08 | 2.21 | 0.990 |
|  | PD | 2.20 | 2.18 | 2.23 | 2.14 × 10^-5^ |
|  | MND | 2.13 | 2.03 | 2.24 | 0.852 |
|  | MG | 2.15 | 2.04 | 2.25 | 0.955 |
|  | Control | 2.14 | 2.14 | 2.15 |  |

*Except for age, t-tests were performed after log transformation

|  | AD | | PD | | MND | | MG | | Control | |
| --- | --- | --- | --- | --- | --- | --- | --- | --- | --- | --- |
|  | Freq | % | Freq | % | Freq | % | Freq | % | Freq | % |
| **Ethnic background** | | | | | | | | | | |
| African | 0 | 0.00 | 2 | 0.21 | 0 | 0.00 | 0 | 0.00 | 984 | 0.85 |
| Any other Asian background | 0 | 0.00 | 3 | 0.32 | 0 | 0.00 | 0 | 0.00 | 479 | 0.41 |
| Any other Black background | 0 | 0.00 | 0 | 0.00 | 0 | 0.00 | 0 | 0.00 | 22 | 0.02 |
| Any other mixed background | 1 | 0.66 | 0 | 0.00 | 0 | 0.00 | 0 | 0.00 | 234 | 0.20 |
| Any other white background | 3 | 1.97 | 23 | 2.43 | 2 | 3.08 | 2 | 3.45 | 4390 | 3.78 |
| Asian or Asian British | 0 | 0.00 | 0 | 0.00 | 0 | 0.00 | 0 | 0.00 | 9 | 0.01 |
| Bangladeshi | 0 | 0.00 | 0 | 0.00 | 0 | 0.00 | 0 | 0.00 | 53 | 0.05 |
| Black or Black British | 0 | 0.00 | 0 | 0.00 | 0 | 0.00 | 0 | 0.00 | 5 | 0.00 |
| British | 136 | 89.47 | 864 | 91.33 | 59 | 90.77 | 53 | 91.38 | 101087 | 87.10 |
| Caribbean | 1 | 0.66 | 5 | 0.53 | 1 | 1.54 | 1 | 1.72 | 1018 | 0.88 |
| Chinese | 1 | 0.66 | 2 | 0.21 | 1 | 1.54 | 0 | 0.00 | 566 | 0.49 |
| Do not know | 0 | 0.00 | 0 | 0.00 | 0 | 0.00 | 0 | 0.00 | 55 | 0.05 |
| Indian | 3 | 1.97 | 8 | 0.85 | 0 | 0.00 | 0 | 0.00 | 1408 | 1.21 |
| Irish | 5 | 3.29 | 23 | 2.43 | 0 | 0.00 | 1 | 1.72 | 3053 | 2.63 |
| Mixed | 0 | 0.00 | 0 | 0.00 | 0 | 0.00 | 0 | 0.00 | 13 | 0.01 |
| Other ethnic group | 1 | 0.66 | 5 | 0.53 | 0 | 0.00 | 1 | 1.72 | 1231 | 1.06 |
| Pakistani | 0 | 0.00 | 1 | 0.11 | 0 | 0.00 | 0 | 0.00 | 430 | 0.37 |
| Prefer not to answer | 1 | 0.66 | 4 | 0.42 | 1 | 1.54 | 0 | 0.00 | 400 | 0.34 |
| White | 0 | 0.00 | 3 | 0.32 | 0 | 0.00 | 0 | 0.00 | 115 | 0.10 |
| White and Asian | 0 | 0.00 | 3 | 0.32 | 0 | 0.00 | 0 | 0.00 | 227 | 0.20 |
| White and Black African | 0 | 0.00 | 0 | 0.00 | 1 | 1.54 | 0 | 0.00 | 118 | 0.10 |
| White and Black Caribbean | 0 | 0.00 | 0 | 0.00 | 0 | 0.00 | 0 | 0.00 | 160 | 0.14 |
| **Prospective memory result (first visit)** | | | | | | | | | | |
| Correct recall on first attempt | 25 | 42.37 | 222 | 71.61 | 20 | 76.92 | 16 | 84.21 | 28604 | 78.35 |
| Correct recall on second attempt | 18 | 30.51 | 60 | 19.35 | 6 | 23.08 | 2 | 10.53 | 6319 | 17.31 |
| Instruction not recalled, either skipped or incorrect | 16 | 27.12 | 28 | 9.03 | 0 | 0.00 | 1 | 5.26 | 1584 | 4.34 |
| **Prospective memory result (second visit)** | | | | | | | | | | |
| Correct recall on first attempt | 8 | 53.33 | 48 | 75.00 | 6 | 75.00 | 1 | 50.00 | 1171 | 86.48 |
| Correct recall on second attempt | 3 | 20.00 | 16 | 25.00 | 2 | 25.00 | 0 | 0.00 | 153 | 11.30 |
| Instruction not recalled, either skipped or incorrect | 4 | 26.67 | 0 | 0.00 | 0 | 0.00 | 1 | 50.00 | 30 | 2.22 |
| **Prospective memory result (third visit)** | | | | | | | | | | |
| Correct recall on first attempt | 6 | 35.29 | 51 | 69.86 | 10 | 76.9 | 6 | 100.00 | 4662 | 84.17 |
| Correct recall on second attempt | 4 | 23.53 | 16 | 21.92 | 2 | 15.38 | 0 | 0.00 | 662 | 11.95 |
| Instruction not recalled, either skipped or incorrect | 7 | 41.18 | 6 | 8.22 | 1 | 7.69 | 0 | 0.00 | 215 | 3.88 |
