# Supplementary material for: Machine Learning Analysis Reveals Biomarkers for the Detection of Neurological Diseases
Source: Front Mol Neurosci. 2022 May 31;15:889728. doi: 10.3389/fnmol.2022.889728 (PMC9194858; doi:10.3389/fnmol.2022.889728)
Supplement: Supplementary file 5 [file Table_3.DOCX]

Supplementary Table 3A. Prediction of training and test sets with the multinomial model

| Training set | | | | | | |
| --- | --- | --- | --- | --- | --- | --- |
|  | Actual diagnosis | | | | |  |
| Predicted diagnosis | AD | PD | MND | MG | Control | **Totals** |
| AD | 5 | 1 | 0 | 0 | 3 | **9** |
| PD | 13 | 170 | 7 | 5 | 8 | **203** |
| MND | 0 | 0 | 0 | 0 | 0 | **0** |
| MG | 0 | 0 | 0 | 1 | 0 | **1** |
| Control | 8 | 59 | 5 | 5 | 565 | **642** |
| **Totals** | **26** | **230** | **12** | **11** | **576** | **855** |
| Test set | | | | | | |
|  | Actual diagnosis | | | | |  |
| Predicted diagnosis | AD | PD | MND | MG | Control | **Totals** |
| AD | 1 | 0 | 1 | 0 | 0 | **2** |
| PD | 8 | 75 | 2 | 3 | 1 | **89** |
| MND | 0 | 1 | 0 | 0 | 0 | **1** |
| MG | 0 | 0 | 0 | 1 | 0 | **1** |
| Control | 2 | 22 | 2 | 1 | 246 | **273** |
| **Totals** | **11** | **98** | **5** | **5** | **247** | **366** |

Supplementary Table 3B. Prediction of training and test sets with the multinomial model excluding demographic measures

| Training set | | | | | | |
| --- | --- | --- | --- | --- | --- | --- |
|  | Actual diagnosis | | | | |  |
| Predicted diagnosis | AD | PD | MND | MG | Control | **Totals** |
| AD | 1 | 2 | 0 | 0 | 0 | **3** |
| PD | 8 | 150 | 7 | 6 | 3 | **174** |
| MND | 0 | 0 | 0 | 0 | 0 | **0** |
| MG | 0 | 0 | 0 | 0 | 0 | **0** |
| Control | 17 | 78 | 6 | 5 | 579 | **685** |
| **Totals** | **26** | **230** | **13** | **11** | **582** | **862** |
| Test set | | | | | | |
|  | Actual diagnosis | | | | |  |
| Predicted diagnosis | AD | PD | MND | MG | Control | **Totals** |
| AD | 1 | 0 | 0 | 0 | 0 | **1** |
| PD | 8 | 65 | 2 | 2 | 1 | **78** |
| MND | 0 | 1 | 0 | 0 | 0 | **1** |
| MG | 0 | 0 | 0 | 0 | 0 | **0** |
| Control | 2 | 32 | 3 | 3 | 248 | **288** |
| **Totals** | **11** | **98** | **5** | **5** | **249** | **368** |
