# Supplementary material for: Machine Learning Analysis Reveals Biomarkers for the Detection of Neurological Diseases
Source: Front Mol Neurosci. 2022 May 31;15:889728. doi: 10.3389/fnmol.2022.889728 (PMC9194858; doi:10.3389/fnmol.2022.889728)
Supplement: Supplementary file 6 [file Table_4.DOCX]

Supplementary Table 4A. Leave-one-out cross validation of the multinomial model

| Variable left out | Accuracy |
| --- | --- |
| None | 0.8825 |
| Age when attended assessment centre | 0.8579 |
| Alanine aminotransferase | 0.8825 |
| Albumin | 0.8798 |
| Apolipoprotein A | 0.8770 |
| Calcium | 0.8825 |
| Cholesterol | 0.8798 |
| Cystatin C | 0.8770 |
| Ethnic background | 0.8798 |
| LDL direct | 0.8770 |
| Mean time to correctly identify matches | 0.8770 |
| Microalbumin in urine | 0.8825 |
| Phosphate | 0.8825 |
| Prospective memory result (first visit) | 0.7596 |
| Prospective memory result (second visit) | 0.8825 |
| Sodium in urine | 0.8770 |
| Testosterone | 0.8699 |

Supplementary Table 4B. Leave-one-out cross validation of the multinomial model when excluding demographic measures

| Variable left out | Accuracy |
| --- | --- |
| None | 0.8533 |
| Alkaline phosphatase | 0.8533 |
| Glucose | 0.8533 |
| Mean time to correctly identify matches | 0.8505 |
| Microalbumin in urine | 0.8533 |
| Phosphate | 0.8533 |
| Prospective memory result (first visit) | 0.6685 |
| Prospective memory result (third visit) | 0.8505 |
| Testosterone | 0.8505 |
| Total bilirubin | 0.8505 |
