# Supplementary material for: Machine Learning Analysis Reveals Biomarkers for the Detection of Neurological Diseases
Source: Front Mol Neurosci. 2022 May 31;15:889728. doi: 10.3389/fnmol.2022.889728 (PMC9194858; doi:10.3389/fnmol.2022.889728)
Supplement: Supplementary file 7 [file Table_5.DOCX]

Supplementary Table 5A. Coefficients of the multinomial generalised linear model.

|  |  |  |  |  |  |  |  |  |
| --- | --- | --- | --- | --- | --- | --- | --- | --- |
|  | **Intercept** | **Age when attended assessment centre [y]** | **Alanine aminotransferase [U/L]** | **Albumin [g/L]** | **Apolipoprotein A [g/L]** | **Calcium [mmol/L]** | **Cholesterol [mmol/L]** | **Cystatin C [mg/L]** |
| AD | -42.91 | 0.24 | -8.45 × 10^-3^ | 0.16 | -3.70 | 3.18 | 1.91 | -0.81 |
| PD | 7.82 | 0.17 | -8.71 × 10^-3^ | 0.05 | -1.82 | 0.15 | 0.00 | 0.78 |
| MND | -1.34 | 0.13 | 4.76 × 10^-3^ | -0.05 | -2.53 | 2.37 | 2.19 | -0.62 |
| MG | -20.01 | 0.10 | 5.81 × 10^-3^ | -0.11 | -0.60 | 1.48 | 1.82 | 4.64 |
|  | **Ethnic background** | | | | | | | |
|  | **African** | **British** | **Caribbean** | **Chinese** | **Indian** | **Irish** | **White and Asian** | **Any other Asian background** |
| AD | -16.51 | 57.11 | -31.42 | 61.05 | -12.45 | 57.24 | -13.62 | -27.57 |
| PD | 29.52 | 28.88 | 27.92 | -16.01 | 29.73 | 29.21 | 30.89 | -23.03 |
| MND | -8.03 | 37.11 | -14.60 | -4.80 | -5.56 | -23.95 | -11.28 | -12.03 |
| MG | -2.45 | 54.63 | -5.05 | 0.42 | 0.17 | -17.48 | -9.75 | -0.60 |
|  | **Ethnic background** | | |  |  |  |  | **Prospective memory result (first visit)** |
|  | **Any other white background** | **Other ethnic group** | **Prefer not to answer** | **LDL direct [mmol/L]** | **Mean time to correctly identify matches [ms]** | **Microalbumin in urine [mg/L]** | **Phosphate [mmol/L]** | **Correct recall on first attempt** |
| AD | -28.14 | -38.01 | -20.28 | -2.84 | 4.76 × 10^-3^ | -3.84 × 10^-3^ | -0.11 | -45.39 |
| PD | 28.18 | 10.05 | -27.68 | -1.01 | 8.34 × 10^-4^ | 9.75 × 10^-4^ | -0.66 | -46.37 |
| MND | 38.48 | -32.02 | 40.74 | -3.70 | 2.78 × 10^-3^ | 1.98 × 10^-3^ | -3.00 | -45.38 |
| MG | -16.76 | -16.96 | -1.95 | -3.92 | 2.47 × 10^-3^ | -3.36 × 10^-5^ | -0.82 | -45.15 |
|  | **Prospective memory result (first visit)** | | **Prospective memory result (second visit)** | |  |  |  |  |
|  | **Correct recall on second attempt** | **Instruction not recalled, either skipped or incorrect** | **Correct recall on first attempt** | **Correct recall on second attempt** | **Sodium in urine [mmol/L]** | **Testosterone [nmol/L]** |  |  |
| AD | -45.21 | -43.30 | 2.89 | -29.00 | 0.0113 | 0.0049 |  |  |
| PD | -46.22 | -46.54 | 0.96 | -30.93 | 0.0108 | 0.0187 |  |  |
| MND | -45.21 | -87.36 | 3.67 | -72.33 | 0.0062 | 0.0247 |  |  |
| MG | -45.79 | -75.11 | -40.22 | -72.16 | 0.0125 | 0.0171 |  |  |

Supplementary Table 5B. Coefficients of the multinomial generalised linear model when trained without demographics data.

|  | **Intercept** | | **Alkaline phosphatase [U/L]** | | **Glucose [mmol/L]** | **Mean time to correctly identify matches [ms]** | **Microalbumin in urine [mg/L]** | **Phosphate [mmol/L]** |
| --- | --- | --- | --- | --- | --- | --- | --- | --- |
| AD | 16.45 | | 1.42 × 10^-2^ | | -0.36 | 6.78 × 10^-3^ | -1.78 × 10^-3^ | -0.26 |
| PD | 17.95 | | 8.27 × 10^-3^ | | 0.26 | 3.29 × 10^-3^ | 1.62 × 10^-3^ | -0.25 |
| MND | 15.12 | | 1.84 × 10^-2^ | | 0.28 | 4.77 × 10^-3^ | 6.73 × 10^-3^ | -1.97 |
| MG | 14.52 | | -9.16 × 10^-3^ | | 0.43 | 4.65 × 10^-3^ | -3.41 × 10^-2^ | 0.37 |
|  | **Prospective memory result (first visit)** | | | | | **Prospective memory result (third visit)** | | |
|  | **Correct recall on first attempt** | | **Correct recall on second attempt** | | **Instruction not recalled, either skipped or incorrect** | **Correct recall on first attempt** | **Correct recall on second attempt** | **Instruction not recalled, either skipped or incorrect** |
| AD | -22.83 | | -22.33 | | -21.06 | 1.18 | -6.22 | -22.26 |
| PD | -23.91 | | -23.65 | | -24.46 | 0.09 | -9.29 | -12.81 |
| MND | -23.52 | | -22.92 | | -61.28 | 0.97 | -26.32 | -26.97 |
| MG | -22.90 | | -23.70 | | -22.92 | 1.33 | -26.67 | -36.48 |
|  | **Testosterone [mmol/L]** | **Total bilirubin [µmol/L]** | |  |  |  |  |  |
| AD | 9.76 × 10^-3^ | -0.098 | |  |  |  |  |  |
| PD | 5.31 × 10^-2^ | -0.018 | |  |  |  |  |  |
| MND | 8.91 × 10^-3^ | -0.023 | |  |  |  |  |  |
| MG | 1.09 × 10^-1^ | -0.088 | |  |  |  |  |  |
