# Supplementary material for: Machine Learning Analysis Reveals Biomarkers for the Detection of Neurological Diseases
Source: Front Mol Neurosci. 2022 May 31;15:889728. doi: 10.3389/fnmol.2022.889728 (PMC9194858; doi:10.3389/fnmol.2022.889728)
Supplement: Supplementary file 8 [file Table_6.DOCX]

Supplementary Table 6. Shared SNPs identified by genome-wide association studies in at least three neurological diseases.

| Affymetrix SNP ID | dbSNP RS ID | Associated gene | OMIM annotation |
| --- | --- | --- | --- |
| AD + PD + MND + MG (9 SNPs) | | | |
| Affx-6002267 | rs7109780 | GAB2 | N/A |
| Affx-24035197 | rs74357320 | LINC00290 | N/A |
| Affx-37460503 | rs78987863 | ACO1 | N/A |
| Affx-28411577 | N/A | HLA-G | Asthma |
| Affx-35916822 | rs75129440 | SIX1 | Branchiootic syndrome; Deafness |
| Affx-36372749 | rs74725337 | HS6ST1 | Hypogonadotropic hypogonadism |
| Affx-25896904 | rs11954189 | GALNT10 | N/A |
| Affx-11689538 | rs11070930 | ONECUT1 | Amelogenesis imperfecta |
| Affx-28483230 | rs36214709 | HLA-DRB6 | N/A |
| AD + PD + MND (26 SNPs) | | | |
| Affx-13427189 | rs72809485 | ZC3H18 | N/A |
| Affx-37147036 | rs117265661 | LAMA2 | Muscular dystrophy |
| Affx-31386906 | rs72675959 | TRPS1 | Trichorhinophalangeal syndrome |
| Affx-2949815 | rs61847486 | PIP4K2A | N/A |
| Affx-34334471 | rs10868716 | SPATA31C2 | N/A |
| Affx-30906527 | rs75731297 | PTPN12 | Colon cancer |
| Affx-28174149 | rs35018945 | TCP10 | N/A |
| Affx-3592936 | rs12415301 | PCBD1 | Hyperphenylalaninemia |
| Affx-16808519 | rs12624500 | SULF2 | N/A |
| Affx-11960689 | rs7167705 | LOXL1 | Exfoliation syndrome |
| Affx-30291699 | rs73687409 | CHN2 | N/A |
| Affx-28436162 | N/A | DDR1 | N/A |
| Affx-2829210 | rs118164677 | ADARB2 | N/A |
| Affx-12378781 | rs17675037 | SNX29 | N/A |
| Affx-4325871 | rs7125270 | TRIM29 | Orofacial cleft; Cleft lip/palate-ectodermal dysplasia syndrome |
| Affx-2527556 | rs76759472 | GPAM | N/A |
| Affx-11085914 | rs75517467 | SPATA7 | Retinitis pigmentosa; Leber congenital amaurosis |
| Affx-21725390 | rs1351752 | TBL1XR1 | N/A |
| Affx-9625325 | rs11840901 | PCDH9 | N/A |
| Affx-37379166 | rs77875439 | STAU2 | N/A |
| Affx-22420864 | rs67895930 | DOCK3 | N/A |
| Affx-10898469 | rs11624916 | ACOT2 | N/A |
| Affx-13293139 | rs76924295 | CMIP | N/A |
| Affx-35631741 | rs112947130 | OR4A47 | N/A |
| Affx-7393142 | rs60140646 | SLCO1C1 | N/A |
| Affx-29199142 | rs599903 | HTR1E | N/A |
| AD + PD + MG (13 SNPs) | | | |
| Affx-36971881 | rs116542297 | LOC100133050 | N/A |
| Affx-21140237 | rs76149190 | EFCC1 | N/A |
| Affx-36670753 | rs114373863 | ZBTB20 | N/A |
| Affx-18615618 | rs12987681 | DNAJC10 | N/A |
| Affx-26144353 | rs17076822 | HMP19 | N/A |
| Affx-34429450 | rs2571346 | PTCH1 | Holoprosencephaly; Basal cell carcinoma; Basal cell nevus syndrome |
| Affx-36710670 | rs114861488 | BCHE | Apnoea |
| Affx-24011624 | rs57948928 | LINC00290 | N/A |
| Affx-16041342 | rs73543247 | PTPRS | N/A |
| Affx-36393504 | rs77796272 | NR4A2 | N/A |
| Affx-4132155 | rs11226859 | GRIA4 | N/A |
| Affx-32549442 | rs3103852 | XKR9 | Otofaciocervical syndrome; Branchiootic syndrome; Anterior segment anomalies; Branchiootorenal syndrome |
| Affx-14272718 | rs76831832 | FLJ37644 | Campomelic dysplasia |
| AD + MND + MG (16 SNPs) | | | |
| Affx-30422455 | rs117218839 | VPS41 | Wilms tumour susceptibility |
| Affx-29251151 | rs78474144 | MAP3K7 | N/A |
| Affx-11505077 | rs80243908 | TMCO5A | N/A |
| Affx-7921171 | rs4658397 | MAP1LC3C | N/A |
| Affx-12105511 | rs117254097 | KLHL25 | N/A |
| Affx-10143080 | rs12589461 | MIR203 | N/A |
| Affx-28360450 | rs75984220 | BTN3A2 | N/A |
| Affx-36729375 | rs115672303 | LPP | Lipoma; Leukaemia, acute myeloid |
| Affx-29549468 | rs73211904 | ST7 | N/A |
| Affx-36781522 | rs116617775 | GNPDA2 | N/A |
| Affx-33435971 | rs72706624 | PSIP1 | N/A |
| Affx-7824085 | rs77078859 | CHRM3 | Eagle-Barrett syndrome |
| Affx-34329363 | rs72749767 | CTSL3P | N/A |
| Affx-25331745 | rs115455317 | FBXL17 | N/A |
| Affx-33426548 | rs62534834 | SMARCA2 | Nicolaides-Baraitser syndrome |
| Affx-6287573 | rs495344 | NOX4 | N/A |
| PD + MND + MG (6 SNPs) | | | |
| Affx-28184785 | rs79097477 | FRMD1 | N/A |
| Affx-35812286 | rs76152173 | FGF9 | Multiple synostoses syndrome |
| Affx-26957921 | rs77838597 | ANKRD31 | N/A |
| Affx-36511074 | rs79777109 | CDH4 | N/A |
| Affx-7008747 | rs10849798 | SPPL3 | N/A |
| Affx-36416342 | rs79916736 | CALCRL | N/A |
